# Knockdown of AMIGO2 suppresses proliferation and migration through regulating PPAR-γ in bladder cancer

**DOI:** 10.1186/s41065-024-00325-z

**Published:** 2024-07-08

**Authors:** Dali Han, Bin Xiong, Xiangxiang Zhang, Chaohu Chen, Zhiqiang Yao, Hao Wu, Jinlong Cao, Jianpeng Li, Pan Li, Zhiping Wang, Junqiang Tian

**Affiliations:** 1 Department of Urology, Lanzhou University Second Hospital, Key Laboratory of Gansu Province for Urological Diseases, Clinical Center of Gansu Province for Nephro-Urology, Lanzhou University, Lanzhou, Gansu Province China; 2 Department of Oncology, Lanzhou University Second Hospital, Lanzhou, Gansu Province China; 3 Department of Urology, Gansu Provincial Hospital, Lanzhou, Gansu Province China

**Keywords:** *AMIGO2*, *PPAR-γ*, RNA-Seq, TMA, Bladder cancer

## Abstract

**Purpose:**

This study aims to reveal the relationship between *AMIGO2* and proliferation, migration and tumorigenicity of bladder cancer, and explore the potential molecular mechanisms.

**Methods:**

The expression level of *AMIGO2* is measured by qRT-PCR and immunohistochemistry (IHC). Stable *AMIGO2* knockdown cell lines T24 and 5637 were established by lentivirus transfection. Cell Counting Kit (CCK-8 assay) was produced to determine cell proliferation, flow cytometry analysis was utilized to detect cell cycle, and wound healing assay was proceeded to test migration ability of bladder cancer cells. Xenograft mouse model was established for investigating the effect of *AMIGO2* on tumor formation in vivo. The RNA Sequencing technology was applied to explore the underlying mechanisms. The expression level of *PPAR-γ* was measured by Western Blot.

**Results:**

*AMIGO2* was upregulated in bladder cancer cells and tissues. Inhibited expression of *AMIGO2* suppresses cell proliferation and migration. Low *AMIGO2* expression inhibited tumorigenicity of 5637 in nude mice. According to RNA-Seq and bioinformatics analysis, 917 DEGs were identified. The DEGs were mainly enriched in cell–cell adhesion, peroxisome proliferators-activated receptors (PPARs) signaling pathway and some other pathways. *PPAR-γ* is highly expressed in bladder cancer cell lines T24 and 5637, but when *AMIGO2* is knocked down in T24 and 5637, the expression level of *PPAR-γ* is also decreased, and overexpression of *PPAR-γ* could reverse the suppression effect of cell proliferation and migration caused by the inhibition of *AMIGO2*.

**Conclusion:**

*AMIGO2* is overexpressed in bladder cancer cells and tissues. Knockdown of *AMIGO2* suppresses bladder cancer cell proliferation and migration. These processes might be regulated by *PPAR-γ* signaling pathway.

## Introduction

Bladder cancer (BCa) is the 6th most common cancer in male (4.4% of all cancers) and the 10th most common cancer in both sex (3.0% of all) [1]. Urothelial carcinoma of the bladder is a prevalent cancer type that causes approximately 150,000 deaths globally each year [2]. Thus, exploring more treatment options is of utmost importance. Novel approaches are currently surfacing, including cancer vaccines, targeted therapeutic regimens, immune modulation strategies, nanoparticle-mediated therapies, cytotoxic agents, hyperthermia, and gene therapy applications [3]. Among all the therapies, gene therapy stands out as a particularly promising contender. Consequently, the exploration of new targets is important, and in-depth studies of the potential molecular mechanisms underlying the biological behavior of bladder cancer cells would aid the identification of reliable molecular markers for bladder cancer patients.

The adhesion molecule with Ig-like domain 2 (*AMIGO2*) located in the 12th chromosome, precisely within the chromosomal region q13.11, spanning a length of 3175 bp. The protein encoded by *AMIGO2* exhibits amphoteric properties and is characterized by an extracellular domain featuring 6 leucine repeat sequences (LRR), along with an Ig-like domain [4]. *AMIGO2* plays a crucial role in various cellular processes, notably including tumor growth, cell adhesion and migration through collagen of gastric cancer cells [5]. In addition, *AMIGO2* has been identified as a critical determinant in the promotion of melanoma cell proliferation and survival. This effect appears to be facilitated by the acquisition of active cis-acting DNA regulatory elements that are notably absent in Normal Human Melanocytes (NHMs) [6].

The peroxisome proliferator-activated receptor-gamma (*PPAR-γ*) represents a prominent constituent within the hormone-responsive nuclear receptor superfamily. As is reported before, *PPAR-γ* induces antiproliferative and antiangiogenic pathways across various tissue types, thus making it a promising target for downregulation of carcinogenesis [7]. In neuroblastoma, *PPAR-γ* plays a tumor-promoting role. In contrast, in breast cancer cells, *PPAR-γ* inhibits inflammatory response, and it functions as a tumor suppressor. Therefore, the research suggests that the ability of *PPAR-γ* to promote or suppress tumor formation is linked to cell type-specific differences in target genes [8]. In hepatocellular carcinoma, instances of functional impairment stemming from mutations affecting suppressive regulators of *PPAR-γ*, such as histone deacetylase 3 (*HDAC3*) and nuclear hormone corepressor (N-CoR), engender a propensity toward steatosis and aberrant hepatic expansion, ultimately culminating in malignancy in both murine models and human subjects [9, 10]. Therefore, the function of *PPAR-γ* across different cancer types seems to be controversial [11].

In the present study, we explored the biological functions of *AMIGO2* in bladder cancer. Our study illuminated that *AMIGO2* promotes the proliferation, migration and tumorigenicity of BCa cells by regulating *PPAR-γ* pathway. These findings deepened our understanding of the molecular mechanisms underlying the progression of bladder cancer. These results might hold promise for finding potential therapeutic targets of bladder cancer.

## Materials and methods

### Cell culture and tissue specimen collection

The human uroepithelium cell line SV-HUC-1, human bladder cancer cell lines T24 and 5637 were obtained from Chinese Academy of Science, authenticated by STR profiling and tested for mycoplasma contamination. SV-HUC-1 was cultured in F-12 K medium, and T24 and 5637 were cultured in RPMI-1640 medium (Gibco, Thermo Fisher Scientific, USA), supplemented with 10% fetal bovine serum (Pan Biotech, Germany), penicillin and streptomycin (100 IU/ml). All the cells were incubated at 37℃ under a humidified atmosphere with 5% CO_2_.

Bladder cancer tissues and their matched adjacent non-tumor bladder tissues (*n* = 16) were collected from patients who underwent radical cystectomy and received a pathological diagnosis of bladder cancer at the Department of Urology, Lanzhou University Second Hospital. All the samples were promptly harvested post-surgery, fixed in formalin, and subsequently embedded in paraffin. None of the enrolled patients underwent chemotherapy, radiotherapy or adjuvant treatment before surgery. All tissue acquisitions were conducted in accordance with the principles of informed consent, with each patient providing written consent for the utilization of their tissue samples in this research. This project was approved by the Institutional Research Ethics Committee of Lanzhou University Second Hospital (No. 2019A-119).

### Tissue Microarray (TMA) construction and image acquisition

The construction of TMAs was executed in accordance with the method outlined previously [12]. Briefly, tissue blocks were meticulously arranged in the desired order. The selection of the appropriate tool diameter, which in our study was set at 2.0 mm, was a critical step. Subsequently, up to 9 TMA blocks were loaded into the apparatus, and distinct identification labels were assigned to each of the 9 blocks. Establish a TMA layout for each recipient block. Following this, the donor blocks were introduced into the tissue microarrayer, and their placement within each row was determined based on their respective case numbers. Each donor block was then attributed with a unique donor ID. Use the punching tool, this instrument punches a hole from the donor block at the selected region. Cores are then transferred from the donor blocks to the recipient one. After the completion of punching holes in the first round, the process moves on to the next round, following the same steps described before. Repeat this process until all the available donor block have been punched. At this point, the production of TMA is finished.

TMA images were viewed and acquired using panoramic scanning microscope (TissueFAXS Viewer 7.1.6245.119, Austria). A total of 16 pairs of cancer tissues and corresponding normal tissues were included in the cohort. One representative image was meticulously chosen for each patient. All images derived from the TMAs were systematically incorporated into the subsequent stages of image processing analysis.

### H&E (Hematoxylin–eosin) staining

Routine dewaxing and hydration were performed on the tissue slices. After staining with hematoxylin for 5 min, differentiation was carried out using 1% hydrochloric acid ethanol. Following a 10-min staining with eosin, the slices were cleared in xylene, dehydrated in alcohol, and mounted with neutral resin. Imaging was captured and viewed using panoramic scanning microscope (TissueFAXS Viewer 7.1.6245.119, Austria).

### Immunohistochemiscal analysis

An IHC analysis was performed using formalin-fixed, paraffin-embedded tissues as previously described [13]. Briefly, the TMA sections were first deparaffinized in xylene and rehydrated in graded alcohol. For antigen retrieval, tissue slides underwent a process immersion and boiling in a 0.01 M citrate buffer solution for 16 min. Subsequently, these slides were incubated in a humidified chamber with *AMIGO2* antibody (1:250, Santa Cruz, USA) overnight at 4℃. Following the primary antibody incubation, the slides were subjected with biotinylated anti-goat IgG secondary antibody for 15 min at room temperature. Sections were then stained with 3, 3`-diaminobenzidine (DAB) and counterstained with hematoxylin (Solarbio, Peking, China). For the evaluation of IHC staining, the samples were scored based on two parameters. The first parameter considered the proportion of positively stained cells, categorized as follows: 0-none, 1—< 25%, 2—25–50%, 3—50–75%, and 4—75–100%. The second parameter assessed staining intensity, with scores assigned as follows: 0—none, 1—weak, 2—medium and 3—strong. To derive a comprehensive score, these two sub-scores were multiplied. Samples achieving total scores in the range of 0–6 were classified as low expression, while 7–12 were considered as high expression. The patients’ information and IHC Scores are listed in Table 1. The clinicopathologic characteristics of 16 cases are documented in Table 2.

**Table 1:** Patients’ information and IHC scores

Patient No	Age	Gender	TNM	Staining Intensity		Quantity		IHC Score	
				Cancer	Normal	Cancer	Normal	Cancer	Normal
1	67	Male	T1N0M0	weak	strong	25%-50%	>75%	2	12
2	68	Male	T4N0M0	strong	weak	50%-75%	<25%	9	1
3	67	Male	T2bN0M0	strong	medium	>75%	25%-50%	12	4
4	64	Male	T2cN0M0	strong	weak	25%-50%	<25%	6	1
5	73	Female	T2aN0M0	medium	strong	50%-75%	>75%	6	12
6	59	Male	T2aN0M0	strong	medium	>75%	25%-50%	12	4
7	69	Male	T2aN0M1	medium	strong	25%-50%	>75%	4	12
8	60	Male	T2cN0M0	strong	strong	>75%	>75%	12	12
9	76	Male	T2cN0M0	strong	medium	>75%	50%-75%	12	6
10	77	Male	T3N1M0	strong	weak	>75%	<25%	12	1
11	56	Male	T1N0M0	strong	weak	50%-75%	<25%	9	1
12	75	Female	T4N0M0	strong	medium	>75%	>75%	12	8
13	72	Male	T2aN0M1	strong	medium	>75%	50%-75%	12	6
14	70	Female	T3N0M0	weak	weak	<25%	<25%	1	1
15	55	Male	T2cN0M0	strong	weak	>75%	<25%	12	1
16	53	Male	T1N0M0	strong	medium	>75%	50%-75%	12	6

*IHC* Immunohistochemistry

**Table 2:** Clinicopathologic characteristics of 16 cases diagnosed with bladder cancer

Variables	Cases	AMIGO2 expression Number of cases (percentage)		*P*
		Low	High	
Total	16	4	12	0.046^*^
Age				0.074
< 65	6	0 (0%)	6 (100%)	
≥ 65	10	4 (33%)	6 (67%)	
Gender				0.064
Male	13	2 (15%)	11 (85%)	
Female	3	2 (67%)	1 (33%)	
T classification				0.687
T1	3	1 (33%)	2 (67%)	
T2	9	2 (22%)	7 (78%)	
T3	2	1(50%)	1(50%)	
T4	2	0(0%)	2(100%)	
N classification				0.551
N0	15	4 (27%)	11 (73%)	
N1	1	0 (0%)	1 (100%)	
N2	0	0 (0%)	0 (0%)	
N3	0	0 (0%)	0 (0%)	
M classification				0.383
M0	14	3 (21%)	11 (79%)	
M1	2	1 (50%)	1 (50%)	
Pathological grade				0.551
Low grade	1	0 (0%)	1 (100%)	
High grade	15	4 (27%)	11 (73%)	
Vascular invasion				0.182
No	12	4 (33%)	8 (67%)	
Yes	4	0 (0%)	4 (100%)	

*p< 0.05

### Cell transfection

Plasmids carrying *AMIGO2*, *PPAR-γ* and negative control shRNA were constructed. Lentivirus was generated in HEK-293 cells. The plasmid was procured from Shanghai Genechem Co., LTD. Lentivirus plasmid constructed with scramble sequence was used as control. The transfection procedure was executed following the manufacturer’s instructions. Puromycin was introduced at a concentration of 1.5 µg/ml for selection. Cell clones that exhibited stable down-regulation of *AMIGO2* (shAMIGO2) and those maintained as negative control (shCtrl) were selected and expanded within T24 and 5637. Then, *PPAR-γ* was stably overexpressed in shAMIGO2 group and shCtrl group in both cell lines, following the same procedure described before.

### RNA extraction, reverse transcription and quantitative real-time PCR

Total RNAs were extracted from cultured cells using Trizol Reagent (Takara, Japan) according to the manufacturer’s instructions. Conversion to cDNA was achieved through cDNA PrimeScript™ RT Master Mix (Perfect Real Time) (TAKARA, Japan) in reverse transcription PCR instrument (Bio-Rad Laboratories, USA). qRT–PCR was carried out using the CFX Real-Time PCR System (Bio-Rad Laboratories, USA) in a 15-μl reaction volume containing first-strand cDNA, TB Green™ Premix Ex Taq™ II (Tli RNaseH Pluse). The relative expression was calculated using the 2^−ΔΔCt^ method [14]. The transcription level of GAPDH was used as an internal control. The primers used are listed in Table 3.

**Table 3:** The primers selected for qRT-PCR

Gene	Forward	Reverse
GAPDH	5’-TGACTTCAACAGCGACACCCA-3’	5’-CACCCTGTTGCTGTAGCCAAA-3’
AMIGO2	5’-CCTGGGAACCTTTTCAGACTG-3’	5’-GCAAACGATACTGGAATCCACT-3’
PPAR-γ	5’-ACTTCTCCAGCATTTCTACTCC-3’	5’-GGCTCCACTTTGATTGCACT-3’

### Protein extraction and Western Blot

Cells were thawed on ice by ultrasonication in RIPA buffer (Beyotime, China). The resulting homogenates were centrifuged at 4℃, 12000 g, for 15 min, followed by the collection of the supernatants. Protein concentration was measured by BCA Protein Assay Kit (Beyotime, China). Protein isolates were then resolved on SDS-PAGE gel and transferred to polyvinylidene difluoride (PVDF) membranes (Millipore, USA). The membranes were blocked with 5% milk, incubated overnight with the primary antibodies: Anti-AMIGO2 (Santa-Cruz, USA); Anti-PPAR-γ (Abcam, UK); β-Actin (Santa-Cruz, USA), and probed with horseradish peroxidase-conjugated secondary antibody: Anti-Mouse IgG (Santa-Cruz, USA). The blots were then detected using Pierce™ ECL Western Blotting Substrate Kit (Thermo, USA).

### Flow cytometric cell cycle analysis

Cells (1 × 10^6^) were collected, re-suspended in PBS, fixed in 75% ice-cold ethanol, and incubated in propidium iodide (PI, 50 µg/ml; Sigma, USA) in the dark for 20 min. RNA enzyme (100 µg/ml) was added into the system. Cell cycle analysis was performed using the BD LSRII Flow Cytometry System with FACSDiva software (BD Bioscience, Franklin Lakes, USA). The data were analyzed using the Modfit LT software.

### CCK-8

Cells were seeded into 96-well plates (2000 cells/well). Cell proliferation was detected using the Cell Counting Kit-8 (CCK-8, Dojindo Laboratories, Japan). At the specified time point, CCK-8 solution was added to each well and incubated for 3 h. The absorbance was measured at 450 nm with a microplate meter (Tecan infinite, Switzerland).

### Wound-healing assay

3 × 10^5^ T24 or 5637 cells were inoculated into 6-well plates with complete culture medium separately. The confluent monolayer of both cells was scratched with a 1 ml pipette tip. The cells were then washed with PBS three times and kept in the medium (supplemented with 10% FBS) for 72 h. The wound width was recorded at 0 h, 24 h, 48 h and 72 h under a microscope.

### Xenograft mouse model

Nude mice (BALB/c-nu, female, 4–6 weeks, 16-18 g) were bred and housed in AAALAC-accredited specific pathogen-free rodent facilities. Mice were accommodated in sterilized and ventilated microisolator cages, receiving ample autoclaved commercial chow and sterile water. The mice were randomly divided into two groups (*n* = 8) and housed in four cages (4 mice per cage). Cage size: 310 × 230 × 160 mm. The environmental conditions were rigorously controlled. Ambient temperature was maintained within the range of 20–25℃, with humidity levels sustained between 40 and 70%. Lighting conditions followed a 12-h light–dark cycle. The overall living conditions were stable. Tumorigenicity was determined by subcutaneously injection of 5637 cells into the flanks of female nude mice (5 × 10^6^ cells per site). Tumors were measured every 2 days using an electronic skin caliper, capturing the longest width and length. Tumor volumes were calculated employing the formula: V = π/6 × largest diameter × smallest diameter^2^ [15]. No blinding was done. All mouse experiments were conducted with standard operating procedures approved by the University Committee on the Use and Care of Animals at Lanzhou University Second Hospital (Approval No. D2019-171).

### RNA-Seq data analysis

Total RNAs were extracted from both shAMIGO2 and shCtrl samples. We employed RNA-Seq technology (Novogene, China) for the identification of DEGs (Differentially Expressed Genes) [16]. In brief, the raw fastq data generated from RNA-seq were initially subjected to trimming using Trimmomatic (V0.35). Subsequently, the trimmed reads underwent alignment to the human reference genome (NCBI GRCh38) utlizing TopHat (Version 2.0.12) with default parameter settings. The resulting aligned bam files were then processed using Cufflinks (Version 2.2.1) for gene quantification. For an additional layer of analysis, reads were also mapped to ERCC (External RNA Controls Consortium) transcripts and quantified using TopHat (Version 2.0.12) and Cufflinks (Version 2.1.1) with default parameter settings. Genes meeting the threshold of FPKM ≥ 1 (Fragments Per Kilobase of transcript per Million mapped reads) across all samples were included in the subsequent analysis to identify DEGs. We could upload the data from RNA-Seq to a public repository if necessary.

### Data analysis and DEGs identification

The sequencing dataset underwent normalization and analysis through the DESeq package (Version 1.10.1). The criteria of FDR < 0.01 (False Discovery Ratio) and |logFC|> 2 (Fold Change) was set as the threshold. Visualization of the dataset was conducted using the R programming software, generating both a volcano plot and a heat map. The most upregulated 100 genes and downregulated 100 genes were chosen for the heat map.

### Biological function and pathway enrichment analysis

Through calculating the corresponding topological overlap, genes positively associated with *AMIGO2* were found out and subjected to gene ontology (GO) analysis (GOSeq, Release2.12) to determine clusters of DEGs with enriched molecular functions. Kyoto encyclopedia of genes and genomes (KEGG) pathway analysis was performed via the “clusterProfiler” package in R/Bioconductor software to acquire the enriched biological process and KEGG pathway. *p* < 0.05 and counts ≥ 4 were considered significant.

### Module analysis of protein–protein interaction (PPI) network

Search Tool for the Retrieval of Interacting Genes (STRING) database (http://www.string-db.org/) was applied to acquire PPI information for the DEGs. Cytoscape software (3.7.2) was applied to visualize the PPI network. The top DEGs with a high degree of connectivity in the PPI network were selected to discuss their function and effect on bladder cancer.

### Kaplan–Meier survival analysis

OS survival data of TCGA samples was downloaded from the Xena database (https://xenabrowser.net/) to study the prognostic significance of hub genes. Kaplan–Meier method was applied for survival analysis with packages “survival” and “survminer”.

### Statistical analysis

All statistical analyses were carried out using IBM SPSS Statistics 20 (SPSS Inc., Chicago, IL, USA). The two-tailed Student’s t-test was used to evaluate statistical differences between two groups. The correlation between *AMIGO2* expression level and patients’ clinicopathological characteristics were analyzed using Chi-squared test. The survival curve was described utilizing the Kaplan–Meier plot. *p < 0.05; **p < 0.01; and ***p < 0.001 were considered statistically significant.

## Results

### *AMIGO2* is upregulated in bladder cancer cells and tissues

**Fig. 1:**
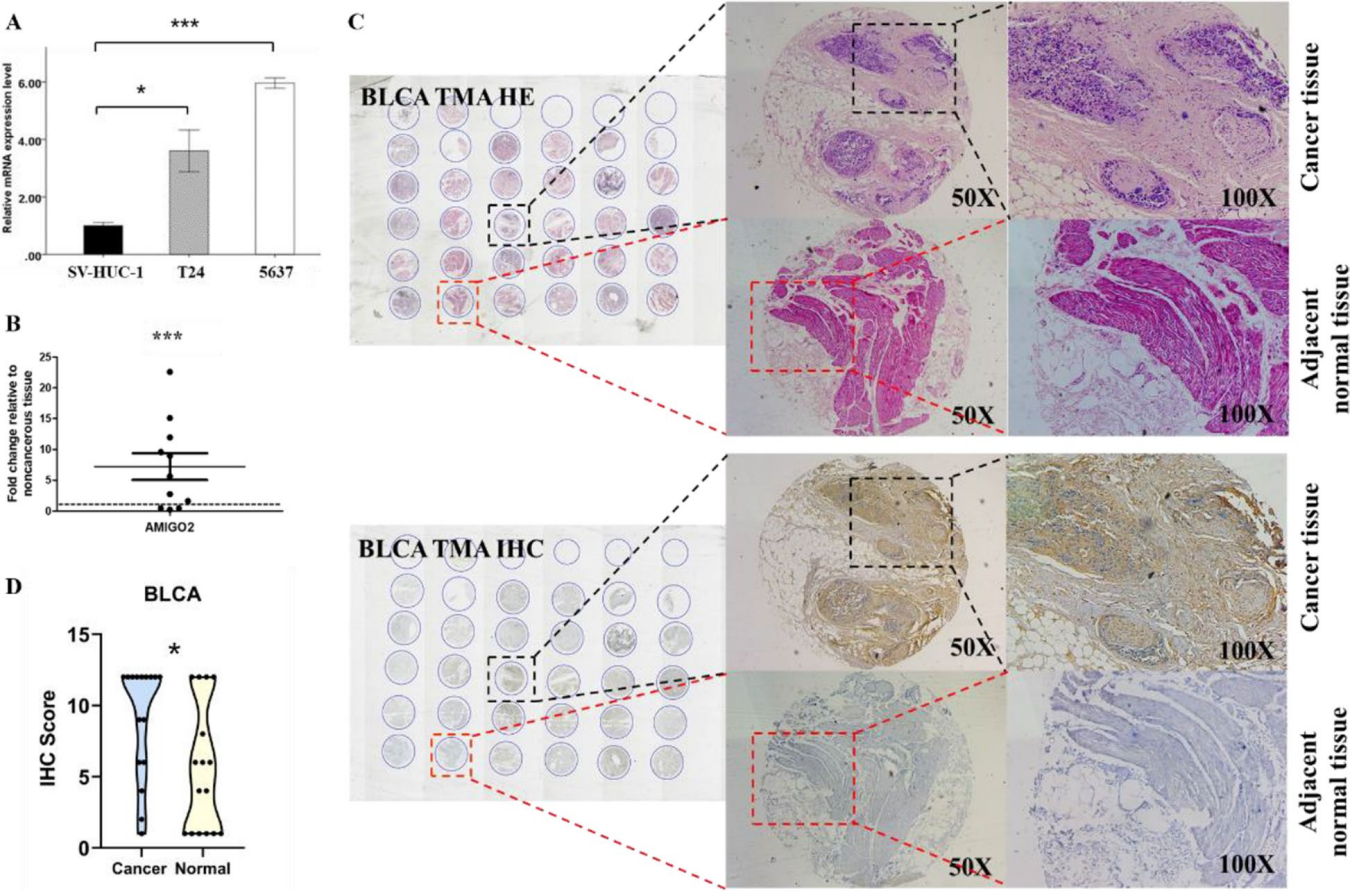
Expression level of *AMIGO2* in bladder cancer cells and tissues. **A** mRNA expression of *AMIGO2* in one normal bladder epithelial cell line SV-HUC-1 and two bladder cancer cell lines: T24 and 5637. **B** The mRNA expression level of *AMIGO2* was examined in cancer tissues (T) and adjacent normal tissues (ANT) (*n* = 11). **C** Representative HE and IHC images of *AMIGO2* protein expression in cancer tissues and the corresponding adjacent normal tissues (*n* = 16). All images were captured at 50 × and 100 × magnification. **D** The violin plots of *AMIGO2* protein expression level in all 16 pairs of tissue samples. Data were based on at least three independent experiments, and shown as mean ± SEM (*p < 0.05, ** p < 0.01, *** p < 0.001). BLCA: Bladder Cancer. TMA: Tissue Microarray

The result of qRT-PCR showed that the *AMIGO2* expression level was elevated in bladder cancer cell lines T24 and 5636 compared with SV-HUC-1 (Fig. 1A). The relative mRNA expression of *AMIGO2* in bladder cancer tissues was significantly higher than that of their matched adjacent normal tissues (*n* = 11) (Fig. 1B). The protein expression level of *AMIGO2* was also up-regulated in bladder cancer tissues compared with the matched adjacent normal tissues (*n* = 16) (Fig. 1C, D). HE and IHC staining were applied to identify. However, as shown in Table 2, according to current evidence, the expression of *AMIGO2* is not associated with age (*P* = 0.074), gender (*P* = 0.064), TNM classification (*P* = 0.687; *P* = 0.551 and *P* = 0.383), pathological grade (*P* = 0.551) or vascular invasion (*P* = 0.182).

### Inhibition of *AMIGO2* suppresses proliferation and migration in vitro

**Fig. 2:**
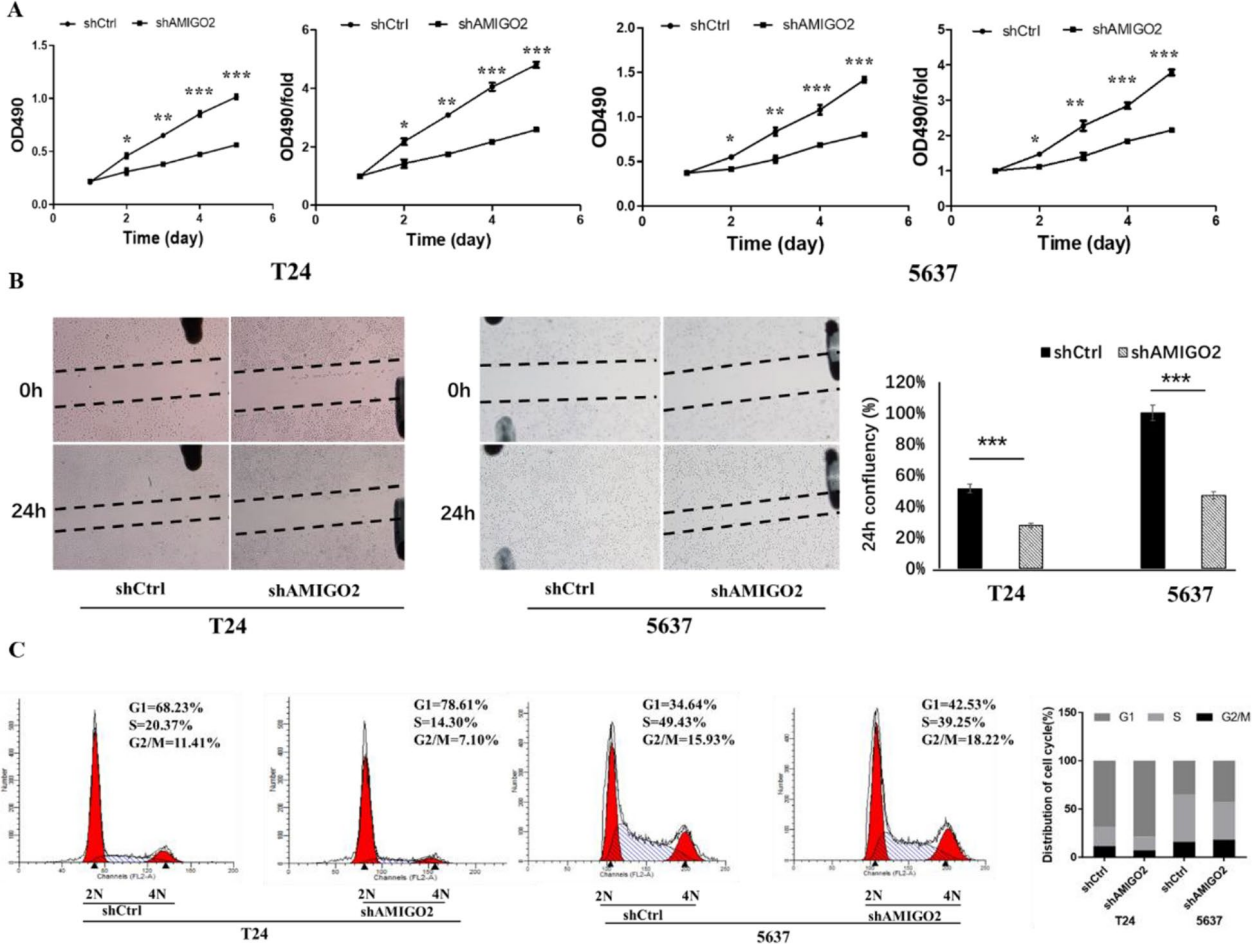
Knockdown of *AMIGO2* suppresses proliferation, migration and cell cycle in bladder cancer. **A** Effects of shAMIGO2 and shCtrl on the proliferation of bladder cancer cell lines T24 and 5637, detected by CCK-8. **B** Effects of shAMIGO2 and shCtrl on the migration BCa cells, assessed by wound healing assay. **C** Effects of shAMIGO2 and shCtrl on cell cycle of the indicated cells, analyzed by flow cytometry. Bars represent the mean ± SEM of three independent experiments. * *p* < 0.05, ** *p* < 0.01, *** *p* < 0.001

CCK-8 and wound healing assay showed that suppression of *AMIGO2* decreased the proliferation and migration capacity of T24 and 5637 cells in shAMIGO2 group compared with shCtrl group (Fig. 2A, B). Flow cytometry displayed an increase in the percentage of cells in G1/G0 phase and a decrease in the percentage of cells in S phase (Fig. 2C). All these results supported that downregulation of *AMIGO2* reduces the proliferation and migration and induces G1 phase cell cycle arrest.

### Inhibition of *AMIGO2* reduces tumorigenicity of bladder cancer cells in vivo

**Fig. 3:**
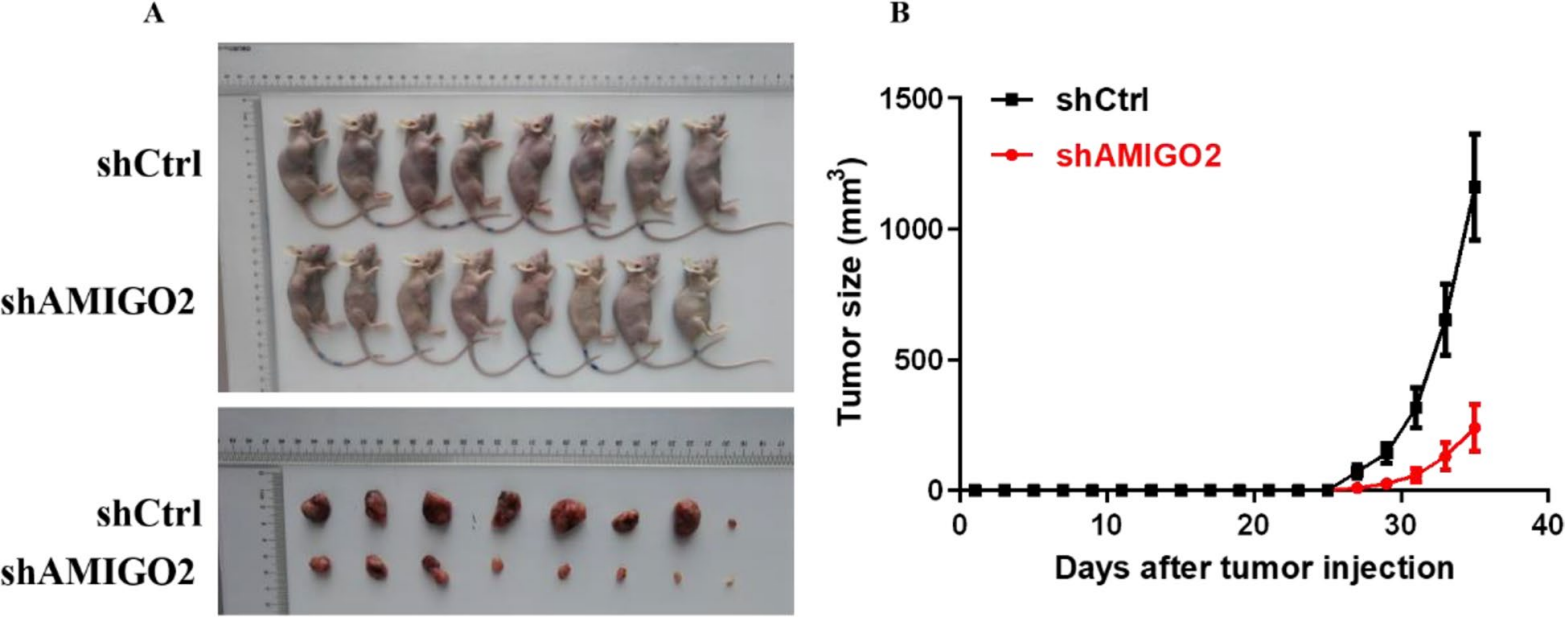
Knockdown of *AMIGO2* inhibits tumorigenicity of bladder cancer cells in vivo. **A** The image of nude mice and tumors. **B** The volume of tumors, detected for 36 days. The results are presented as the means ± SEM of values (*n* = 8). Statistical significance was calculated using the Student’s t-tests. (*p < 0.05, **p < 0.01, ***p < 0.001)

The growth of tumors derived from the shAMIGO2 group was prominently suppressed compared with the shCtrl group after 5637 injection (*n* = 8), detected for 36 days (Fig. 3A, B). These results indicated that inhibition of *AMIGO2* reduces bladder cancer cell growth and tumorigenicity in vivo, which was consistent with the in vitro results.

### Identification of differentially expressed genes (DEGs) and molecular function and pathway enrichment analysis

**Fig. 4:**
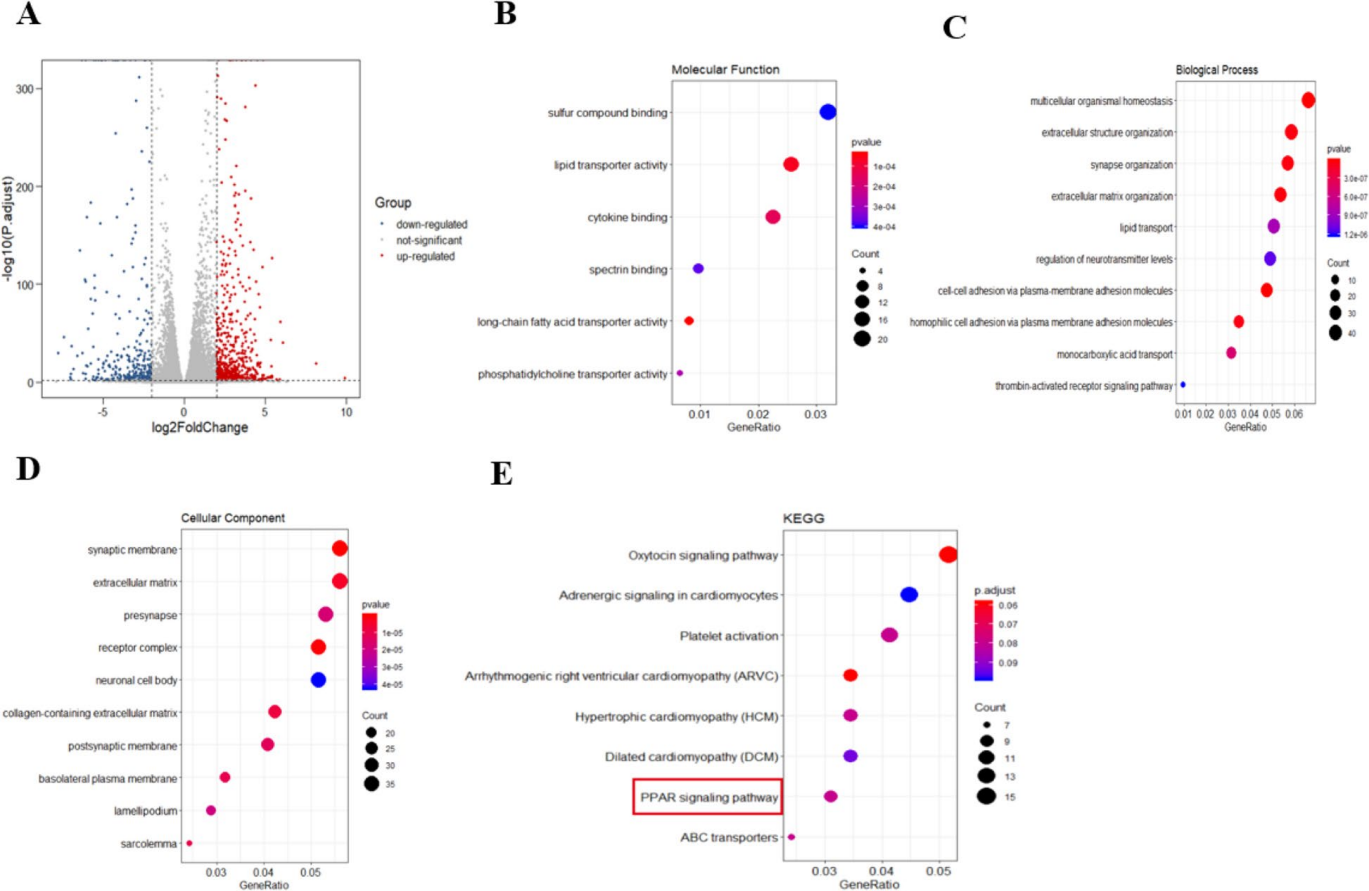
Differentially expressed genes (DEGs) and pathways analyzed by bioinformatics. **A** Identification of differentially expressed genes. Volcano plot of gene expression profiles. Red/blue symbols classify the upregulated/downregulated genes according to the criteria: |logFC|> 2 and adjusted *p*-value. **B** Molecular function, which were significantly enriched in lipid transporter activity, cytokine binding and long-chain fatty acid transporter activity; **C** Biological process, which were significantly enriched in multicellular organismal homeostasis, extracellular structure organization and synapse organization; **D** Cellular component, which were significantly enriched in synaptic membrane, extracellular matrix and receptor complex. **E** KEGG pathways in which DEGs were significantly enriched. There were 8 pathways in the relation graph, including oxytocin signaling pathway, adrenergic signaling in cardiomyocytes, platelet activation, *ARVC, HCM, DCM, PPAR* signaling pathway and *ABC* transporters. Size of the dots: Count of DEGs enriched in corresponding GO and KEGG classification

As the volcano plots illustrated, after data integration, gene expression profiles from RNA Sequencing identified 917 differentially expressed genes. Among all the DEGs, 627 genes were upregulated and 290 were downregulated in shAMIGO2 compared with the shCtrl in T24 (Fig. 4A), grounded on the cut-off criteria (|logFC|> 2, *P*adj < 0.01). DEGs were selected for integrated analysis. In order to investigate the molecular function and biology pathways of the DEGs, GO and KEGG analysis were performed. GO (Gene Ontology) includes molecular function, biological process and cellular component (Fig. 4B-D). Enriched KEGG pathways of the DEGs are shown in Fig. 4E, including PPAR signaling pathway and some other pathways.

### Protein–Protein Interaction (PPI) network construction, hub genes and survival analysis

**Fig. 5:**
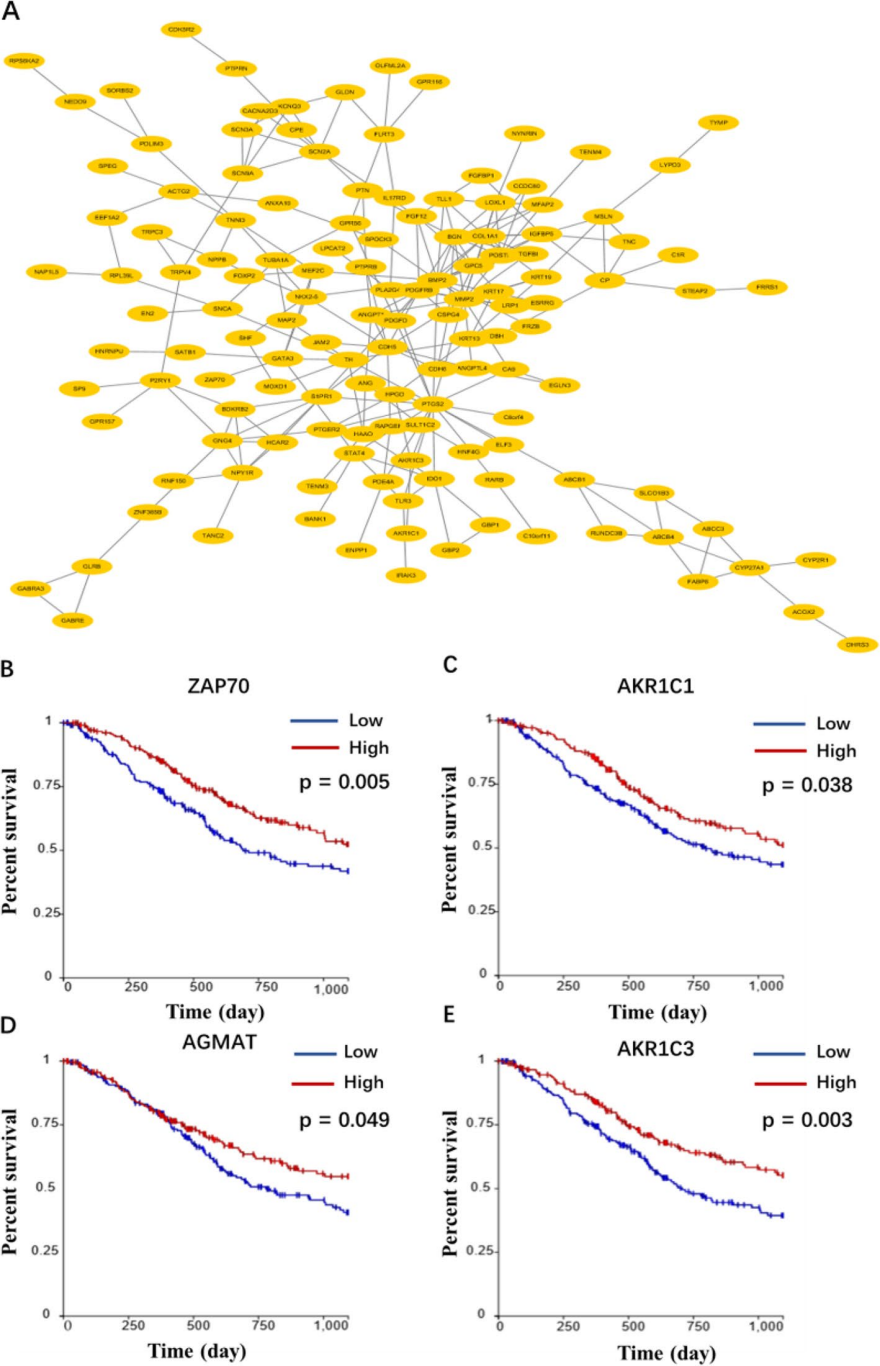
Construction of PPI network and relationship between hub genes and prognosis of bladder cancer. **A** Modules with relatively high score selected from the protein–protein interaction network. The PPI network contains 136 nodes and 234 edges. *ZAP70, AKR1C1, MAP2K6, SCN2A, AGMAT, CBLB, AKR1C3, TLR3, SCN3A* and *AZIN2* were considered as hub genes. **B**-**E** Four genes significantly related to survival. The red line indicates the group with higher expression of this gene, and the blue line indicates the group with lower expression

The PPI network was constructed based on the SRTING database. A total of 174 proteins were obtained from the DEGs, including 136 nodes and 234 edges (Fig. 5A). In the network, nodes with top 10 highest degrees were *ZAP70, AKR1C1, MAP2K6, SCN2A, AGMAT, CBLB, AKR1C3, TLR3, SCN3A and AZIN2*. These genes were considered as hub genes. The information of the 10 hub genes is shown in Table 4, including full gene names and primary functions. Total 10 hub genes were obtained from PPI network. The Kaplan–Meier survival analysis (Fig. 5B-E) shows that four hub genes associated with survival were *ZAP70, AGMAT, AKR1C1* and *AKR1C3.*

**Table 4:** Functional roles of the 10 hub genes

No	Gene	Full name	Function
1	ZAP70	zeta chain of T cell receptor associated protein kinase 70	An enzyme belongs to the PTK family, functions in T-cell development and lymphocyte activation
2	AKR1C1	aldo–keto reductase family 1 member C1	Catalyze the conversion of aldehydes and ketones to their corresponding alcohols
3	MAP2K6	mitogen-activated protein kinase kinase 6	Involved in many cellular processes such as stress induced cell cycle arrest, transcription activation and apoptosis
4	SCN2A	sodium voltage-gated channel alpha subunit 2	Member of the sodium channel alpha subunit gene family
5	AGMAT	agmatinase	Part of an operon in Escherichia coli, constitutes the primary pathway of polyamine synthesis from arginine
6	CBLB	Cbl proto-oncogene B	Encodes an E3 ubiquitin-protein ligase
7	AKR1C3	aldo–keto reductase family 1 member C3	Catalyze the conversion of aldehydes and ketones to their corresponding alcohols
8	TLR3	toll like receptor 3	A member of the Toll-like receptor (TLR) family, functions in pathogen recognition and activation of innate immunity
9	SCN3A	sodium voltage-gated channel alpha subunit 3	Member of the sodium channel alpha subunit gene family
10	AZIN2	antizyme inhibitor 2	The protein belongs to the antizyme inhibitor family, works in cell growth and proliferation

### Overexpression of *PPAR-γ *reverted the inhibitory effect of *AMIGO2* in bladder cancer cells

**Fig. 6:**
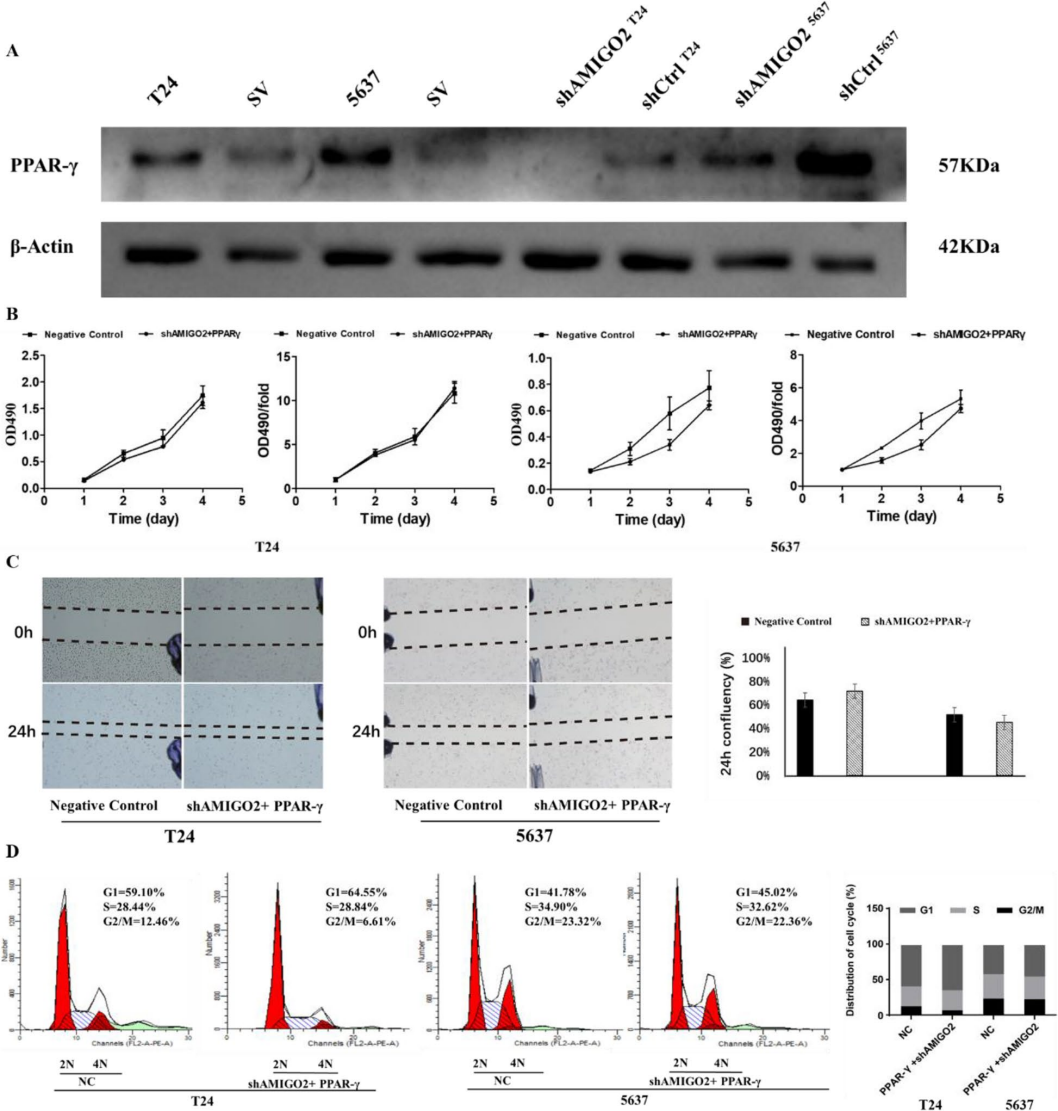
Overexpression of *PPAR-γ* reverted the inhibitory effect of *AMIGO2* in bladder cancer cells. Experimental grouping: shAMIGO2 + PPAR-γ and Negative Control. **A** The expression level of *PPAR-γ* in untreated T24, 5637 and SV-HUC-1 cell lines; the expression level of *PPAR-γ* in shAMIGO2 + PPAR-γ and Negative Control group of bladder cancer cell lines T24 and 5637*.* Measured by Western Blot. β-Actin served as the loading control. **B** Overexpression of *PPAR-γ* reverted the inhibitory effect of *AMIGO2* on proliferation of bladder cancer cells, detected by CCK-8. **C** Overexpression of *PPAR-γ* reverted the inhibitory effect of *AMIGO2* on migration of bladder cancer cells, assessed by wound healing assay. **D** Overexpression of *PPAR-γ* reverted the inhibitory effect of *AMIGO2* on cell cycle of bladder cancer cells, analyzed by flow cytometry

To explore the molecular mechanism of *AMIGO2* function in bladder cancer cells, the target of *AMIGO2* was predicted using bioinformatics analysis. According to the results of RNA-Seq, *PPAR-γ* was identified as the target of *AMIGO2* (Fig. 4E). *PPAR-γ* is overexpressed in bladder cancer cell lines T24 and 5637, but when *AMIGO2* is knocked down in T24 and 5637, the expression level of *PPAR-γ* is also decreased (Fig. 6A). Meanwhile, the results of CCK-8 and wound-healing assay both indicated that overexpression of *PPAR-γ* reverted the inhibitory effect of *AMIGO2* on proliferation, migration and cell cycle of bladder cancer cells (Fig. 6B-D). These results suggested that knockdown of *AMIGO2* suppresses proliferation and migration by regulating *PPAR-γ* in bladder cancer.

## Discussion

Previous researches into various genes associated with malignancies has provided valuable insights into the underlying mechanisms of bladder cancer. Studies have indicated that the aberrant expression of specific genes plays a pivotal role in driving the progression of distinct tumor subtypes. In the present study, we found that *AMIGO2* was upregulated in bladder cancer cells and tissues, and it could promote the proliferation, migration and tumorigenicity. To explore the mechanism of *AMIGO2*-induced cell proliferation, we investigated potential targets of *AMIGO2* and found that *PPAR-γ* is targeted by *AMIGO2* and is essential for the bio-function of bladder cancer.

The unconstrained cellular proliferation observed in cancer is mainly attributed to the cell cycle deregulation [17]. DNA damage targets two cell cycle checkpoints, namely the G1/S and G2/M. DNA damage triggers a regulatory program that enforces cell cycle at a designated checkpoint, either until the damage is repaired or the cells move towards apoptosis [18]. Arresting cells in G0/G1 phase presents an opportunity for certain cells to either undergo repairing or move towards apoptosis. The flow cytometry assay in our study showed that suppression of *AMIGO2* could inhibit its proliferative effects through blockage of cell cycle progression and arrest BCa cells in G1 phase. In many cases, an arrest can lead to senescence or apoptosis [19]. Although few studies have described the relationship between *AMIGO2* and cell cycle before, there are some pathways that regulate cell cycle indirectly enriched by our KEGG analysis, but the particular pathways and molecules are still under research.

GO term enrichment analysis showed that the DGEs were significantly enriched in extracellular matrix, lipid transporter activity and cell–cell adhesion via plasma-membrane adhesion molecules, suggesting that some of the DEGs might be involved in cell adhesion and migration. As stated in previous studies, *PCDH1* (Protocadherin 1) mediated cell–cell adhesion through homotypic interactions [20], *CELSR1* (cadherin EGF LAG seven-pass G-type receptor 1) regulated endothelial adherens junctions and directed cell rearrangements during valve morphogenesis [21], and *PCDHGA3* worked as one of the cell adhesion molecules in human ischemic cardiomyopathy [22]. Cell adhesion participants in stimulating signals that regulate cell migration, cell cycle, and cell survival [23]. Cell adhesiveness is generally reduced across different types of human cancers. *AMIGO2*, as reported before, is also involved in cell adhesion and/or migration [5, 24, 25]. Changes of these molecules might be resulting in the reduction of adhesion between cells and promoting the migration of tumor cells. Correspondingly, the wound healing assay in our study also proved that *AMIGO2* could promote the migration of BCa cells.

Subsequently, we explored the interaction of DEGs. A large and complex interactome network was established, suggesting intricate links among those DEGs. Moreover, 10 hub genes were identified in total, 4 of them were related to survival, namely *ZAP70, AGMAT, AKR1C1* and *AKR1C3. ZAP70* was reported to be involved in the development of chronic lymphocytic leukemia [26]. *AGMAT* could promote the lung adenocarcinoma tumorigenesis by activating the NO-MAPKs-PI3K/Akt pathway [27]. *AKR1C1* was proved to be related with the invasive potential and drug resistance of metastatic bladder cancer cells [28]. *AKR1C3,* comes from the same family as *AKR1C1*, has not been proved to have similar functions as *AKR1C1* in bladder cancer, but it is often overexpressed in prostate cancer tissues and cell lines [29]. Interestingly, *AKR1C3* catalyzes the formation of prostaglandin (PG) F2α and 11β-PGF2α from *PGH2* and *PGD2*, respectively [30]. The *PGF2α* and *11β-PGF2α* can inactivate *PPAR-γ* and has anti-proliferative effects [31].

*PPAR-γ* is a member of the nuclear receptor superfamily, it participates in multiple physiological and pathological processes. Extensive researches have revealed the relationship between *PPAR-γ* and various tumors. However, the expression level and functions of *PPAR-γ* seem to be controversial [11]. Some studies have indicated that *PPAR-γ* acts as a tumor promoter [32, 33]. Downregulation of *PPAR-γ* has been shown to inhibit the growth of cancer cells, suggesting a tumor-promoting effect for *PPAR-γ* [33, 34]. Nevertheless, some other researches illuminated that *PPAR-γ* serves as a tumor inhibitor, *PPAR-γ* suppressed cell growth and invasiveness by blocking cell cycle and stimulating apoptosis and differentiation [7, 35]. In our study, we proved that overexpression of *PPAR-γ* in *AMIGO2*-repressed cells could revert the inhibitory effect of shAMIGO2 on bladder cancer cells. Therefore, we consider *PPAR-γ* as a tumor promoter in bladder cancer.

To the extent of our current understanding, this is the first study determining the very “new” gene *AMIGO2*, which promotes the proliferation, migration and tumorigenicity in BCa. Mechanistically, we identified the *PPAR-γ* as a principal molecular subject to modulation by *AMIGO2* within bladder cancer cell populations. Interestingly, we found that overexpression of *PPAR-γ* in *AMIGO2*-repressed cells could revert the inhibitory effect of shAMIGO2 on bladder cancer cells. As a consequence, it is identified that downregulating *AMIGO2* inhibits bladder cancer cells proliferation and tumorigenicity by suppressing *PPAR-γ*, and that *PPAR-γ* is essential for *AMIGO2*-mediated cell proliferation in bladder cancer.

## Conclusion

*AMIGO2* is overexpressed in bladder cancer cells and tissues. Knockdown of *AMIGO2* suppresses bladder cancer cell proliferation and migration. These processes might be regulated by *PPAR-γ* signaling pathway.

## Data Availability

The datasets used and/or analysed during the current study are available from the corresponding author on reasonable request.
